# How have advances in CT dosimetry software impacted estimates of CT radiation dose and cancer incidence? A comparison of CT dosimetry software: Implications for past and future research

**DOI:** 10.1371/journal.pone.0217816

**Published:** 2019-08-14

**Authors:** Susannah Maxwell, Richard Fox, Donald McRobbie, Max Bulsara, Jenny Doust, Peter O’Leary, John Slavotinek, John Stubbs, Rachael Moorin

**Affiliations:** 1 Health Systems and Health Economics, School of Public Health, Faculty of Health Sciences, Curtin University, Perth, Western Australia, Australia; 2 School of Physics, University of Western Australia, Perth, Western Australia, Australia; 3 School of Physical Sciences, University of Adelaide, Adelaide, South Australia; 4 Faculty of Medicine, Imperial College, London, United Kingdom; 5 Institute for Health and Rehabilitation Research, University of Notre Dame, Fremantle, Western Australia, Australia; 6 Centre for Health Services Research, School of Population Health, Faculty of Medicine, Dentistry and Health Sciences, University of Western Australia, Crawley, Western Australia, Australia; 7 Centre for Research in Evidence-Based Practice Faculty of Health Sciences and Medicine Bond University, Gold Coast, Queensland, Australia; 8 Obstetrics and Gynaecology Medical School, Faculty of Health and Medical Sciences, The University of Western Australia, Perth, Western Australia, Australia; 9 PathWest Laboratory Medicine, QE2 Medical Centre, Nedlands, Western Australia; 10 SA Medical Imaging, SA Health and College of Medicine and Public Health, Flinders University, Adelaide, South Australia, Australia; 11 CanSpeak Australia, Spring Hill, Queensland, Australia; Mizoram University, INDIA

## Abstract

**Objective:**

Organ radiation dose from a CT scan, calculated by CT dosimetry software, can be combined with cancer risk data to estimate cancer incidence resulting from CT exposure. We aim to determine to what extent the use of improved anatomical representation of the adult human body “phantom” in CT dosimetry software impacts estimates of radiation dose and cancer incidence, to inform comparison of past and future research.

**Methods:**

We collected 20 adult cases for each of three CT protocols (abdomen/pelvis, chest and head) from each of five public hospitals (random sample) (January-April inclusive 2010) and three private clinics (self-report). Organ equivalent and effective dose were calculated using both ImPACT (mathematical phantom) and NCICT (voxelised phantom) software. Bland-Altman plots demonstrate agreement and Passing-Bablok regression reports systematic, proportional or random differences between results. We modelled the estimated lifetime attributable risk of cancer from a single exposure for each protocol, using age-sex specific risk-coefficients from the Biologic Effects of Ionizing Radiation VII report.

**Results:**

For the majority of organs used in epidemiological studies of cancer incidence, the NCICT software (voxelised) provided higher dose estimates. Across the lifespan NCICT resulted in cancer estimates 2.9%-6.6% and 14.8%-16.3% higher in males and females (abdomen/pelvis) and 7.6%-19.7% and 12.9%-26.5% higher in males and females respectively (chest protocol). For the head protocol overall cancer estimates were lower for NCICT, but with greatest disparity, >30% at times.

**Conclusion:**

When the results of previous studies estimating CT dose and cancer incidence are compared to more recent, or future, studies the dosimetry software must be considered. Any change in radiation dose or cancer risk may be attributable to the software and phantom used, rather than—or in addition to—changes in scanning practice. Studies using dosimetry software to estimate radiation dose should describe software comprehensively to facilitate comparison with past and future research.

## Introduction

Computed Tomography (CT) scanning provides an essential tool for protecting and improving health[1]. The technology is widely used to diagnose disease, define its extent, assess response to therapy and aid in the planning and conduct of medical procedures and interventions. However, every CT scan delivers a small radiation dose to the body that is potentially carcinogenic. Concerns about the adverse impact of this radiation dose, and a world-wide trend towards increasing collective radiation dose[2, 3] have led to guidelines advising on the indications for CT scanning and radiation dose,[4, 5] as well as epidemiological research on the potential incidence and mortality of cancers resulting from exposure to CT scans within a population.[2, 6–12]

Determining potential cancer incidence as a result of CT scan radiation exposure requires calculations of both the radiation dose to specific organs from a CT scanning protocol, and the risk of cancer as a result of these doses. The former can be obtained from CT scanning protocol data—radiation quantity and the anatomical location of the scan—using ‘Monte Carlo’ calculations. These calculations consider the theoretical path of a very large number of photons entering the body undergoing scattering and absorption interactions with the tissues that they encounter. Results can be reported as ‘absorbed dose’ (in milligrays, mGy) and ‘equivalent’ dose (in milliseiverts, mSv) to specific organs, and effective dose (mSv). The absorbed dose depends on the physical radiation quantity and the absorption properties of the irradiated tissue. The equivalent dose goes further by taking into account the radiation type by applying a weighting factor to the absorbed dose, which in the case of CT scanning is one. The effective dose, as will be discussed in more detail, is a measure of stochastic health risk to the entire body.

Many software programs (ImPACT[13], CT-Expo[14], VirtualDose[15], NCICT[16, 17], WINDOSE[18]) are available which can efficiently calculate absorbed and equivalent organ radiation dose from CT scanning parameters. The estimation of absorbed dose requires reference data on organ mass and an anatomical representation of the human body, known as a “phantom”[19]. Phantoms can be designed to represent an average adult male, female or hermaphrodite (representing organs of both males and females), infants or children. The type of phantom used within dosimetry software programs vary. Phantoms may be based on mathematical models which use quadratic equations to describe organ and body structure, or more advanced voxelised phantoms made up of 3D pixels (or voxels) created from cross sectional medical images, or hybrid phantoms, which are a combination of both. The earlier mathematical phantoms are limited in their ability to describe detailed human anatomy, with organs represented by cylindrical, conical and elliptical and spherical surfaces, and in 2009 the International Commission on Radiological Protection (ICRP) recommended the use of the more anatomically correct voxelised phantoms, specifically ICRP AM and ICRP AF[20], two phantoms representing a reference adult male (AM) and a reference adult female (AF) for the calculation of organ radiation dose.

As the potential health effects from radiation depend not just on the quantity of absorbed radiation but also on how sensitive that organ is to radiation, absorbed and equivalent dose are not sufficient for measuring health risk. Rather, a tissue weighting factor can be applied to each organ specific equivalent dose to take this biologic sensitivity into account, the sum of which is an estimate of the stochastic health risk to the entire body. This measure, known as effective dose (mSv), is an output of dosimetry software. Effective dose is considered an estimate of cancer risk. However, as this measure is not specific to organ, gender or any particular age, its use should be restricted to relative comparisons of radiation exposures across populations. The preferred method for epidemiological studies estimating excess cancer incidence and mortality as a result of CT scanning has been to multiply organ specific equivalent doses with organ-age-sex specific attributable risk coefficients.[21] Attributable risk provides an estimate of the number of cases of cancer among exposed individuals that can be attributed to a unit (mGy) of ionising radiation, that is, how much extra disease has been caused by the radiation exposure, or how much cancer would be prevented if the exposure were eliminated.

Studies investigating the impact of using different software and, mathematical phantoms compared to voxelised or hybrid phantoms, have shown significant deviation in the calculated absorbed, equivalent and effective doses [18, 22–27]. These have been attributed to variation in anatomy between phantoms which lead to the inclusion or exclusion of specific organs and differences in scan length even when using the same anatomical start and end positions, as well as variation in scanner matching methods between software programs.[18] While these studies report the impact of phantom type on the estimates of absorbed dose to specific organs and effective dose, none has considered how differences in the calculated absorbed dose impact estimates of cancer incidence.

The interpretation of epidemiological work often requires comparison with historical or international data to allow commentary on trends and variation over time and/or place. However, in the context of CT dosimetry and cancer risk estimates, the change to voxelised phantoms may impact the ability to make these comparisons. In this study we aim to determine how estimates of radiation dose and cancer incidence have changed with the move to the more anatomically correct voxelised phantoms. We consider to what extent changing the software—and phantom type—used to calculate radiation dose in adults impacts the estimates of 1) effective dose and organ equivalent doses among BEIR VII cancer categories[28] and 2) cancer incidence among those exposed to these radiation doses. The software programs compared are ImPACT[13] version 1.0.4 (developed by the scanner evaluation centre of the United Kingdom National Health Service) which uses a mathematical phantom and NCICT[16, 17] (developed by the National Cancer Institute in the USA) which uses a voxelised phantom.

## Methods

### Data collection

Technical CT scan data were collected for a selection of adult diagnostic CT scanning protocols undertaken at five public hospitals in Western Australia between January 1 and April 30 2010, via the Picture Archiving and Communication System database (PACS). The PACS database includes all scans performed at public hospitals in Western Australia. Data collection has been described in detail previously.[29] In summary, a random sample of 20 cases were collected for each of three protocols 1) abdomen/pelvis (CTA1) 2) chest (CTC1) and 3) head (CTH4) for each of five tertiary and secondary public hospitals, excluding specialist satellite centres. Where less than 20 cases were identified, all cases were retrieved. Another sample of 20 cases from each of three private stand-alone radiology practices was sourced for each protocol from a self-report survey previously reported.[30] Twenty cases per provider were used in this study as this is the standard practice for estimating typical doses delivered by scanning protocols, and exceeds European Guidelines on the collection of dosimetry data for development of dose reference levels for CT which recommends a minimum sample of 10 cases.[31, 32] Technical data parameters were collected for separate scanning sequences for each case and included kilovoltage (kV), milliamperage (mA), tube rotation time, collimation width, pitch, volume weighted CT dose index (CTDIvol), dose-length product (DLP) and scanner model. ImPACT uses a mathematical hermaphrodite phantom “HPA18+” (1.74m, 70kg). This phantom is a version of an earlier mathematical phantom NCRP18+ that has been adapted for use with the ICRP 103 reference data [33]. NCICT uses the voxelised ICRP reference phantoms, ICRP AM and AF which model a reference male (1.78m, 73kg) and a reference female (1.68m, 60kg) respectively. This study was approved by the Western Australia Department of Health Human Research Ethics Committee and the Curtin University Ethics Committee, which exempted the study from requiring individual patient consent. Data did not include any identifying information.

### Organ equivalent dose and effective dose

Each case was subject to one or more scanning acquisitions within a CT protocol. Organ equivalent dose and effective dose were calculated (in mSv) for the total number of scans within the protocol for each case using 1) ImPACT dosimetry software and 2) NCICT dosimetry software. Only helical acquisitions were included in the analysis. The input parameters, body/head filter, the scan start and end locations and kV were required by both the NCICT and ImPACT software. The ImPACT software calculated CTDIvol within the software based on other input parameters, including the scanner model, mA, rotation time, pitch and collimation, whereas NCICT allowed direct input of the CTDIvol collected in the technical data for each case. Another difference was that NCICT used a sex-specific phantom, while ImPACT did not differentiate.

**Fig 1:**
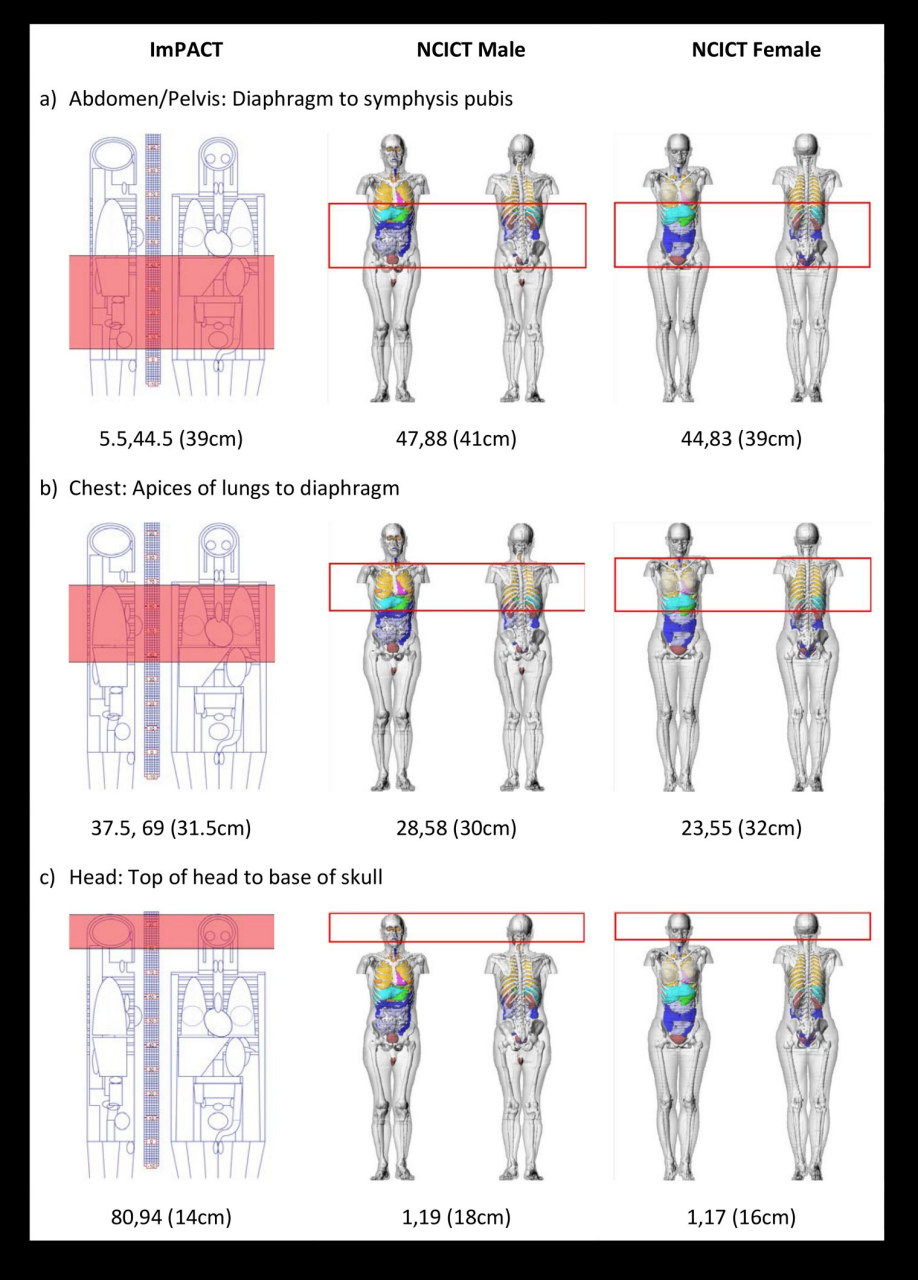
**ImPACT and NCICT phantom scan start end positions for a) abdomen/pelvis b) chest and c) head protocols.** Start and end measurements are indicated below each phantom diagram. Length of scan is shown in brackets. Phantom images are screenshots from ImPACT and NCICT software adapted to show stop start locations for the three protocols.

Our primary objective was to compare the dosimetry software output for ImPACT and NCICT. Therefore, we used the same anatomical start and end positions for each analysis, irrespective of the individual CT scan length data for each case. Using the same anatomical landmarks enabled a more consistent scan position and allowed us to adapt the position for the sex-specific analysis. We identified the appropriate sex-specific anatomical start and end positions on the NCICT phantom, and matched these as closely as possible on the ImPACT software (hermaphrodite) (Fig 1). Identifying a consistent start-end location between phantoms was challenging due to the variation between phantom anatomy and positioning of the body, particularly the head. Scan start-end position and phantoms are shown in Fig 1. These anatomical positions were consistent with the typical anatomical reference start-end positions of these types of scans as identified by local medical imaging technologists[30]. CTDIvol in conjunction with scan start-end position and kV provided all the technical data necessary for the calculation of organ radiation dose. While the NCICT software based their dosimetry calculation on the CTDIvol, we were unable to input the CTDIvol directly into the ImPACT software. Rather, the ImPACT software calculated CTDIvol from other technical input. This resulted in some variation in the CTDIvol value used for the dosimetry estimate. In order to normalise the CTDIvol between the software programs and for CTDIvol to be consistent with that reported in the dataset, we were required to manipulate the technical input parameters in the ImPACT software.

ImPACT and NCICT software both generate organ specific absorbed dose (mGy), equivalent dose (mSv) and effective dose (mSv). We present the organ specific results for those organs included in the BEIR VII risk tables to calculate total cancer incidence. These BEIR VII categories include leukaemia (which appears as “bone marrow” in ImPACT and “active marrow” in NCICT), and the category “other”. “Other” is not a category provided by ImPACT or NCICT. For “Other” we used the mean of the median doses for organs not named in the BEIR VII LAR tables but which were included in the remainder organs listed by ICRP 103 [2] (S1 Table). This is an approximation and assumes each organ contributes equally to the risk. NCICT software provided sex-specific results. For the reporting of effective dose, we averaged the male and female effective dose for comparison with the ImPACT hermaphrodite result. As a previous study had shown tube voltage affected the ratio of the organ and effective doses calculated by mathematical phantom software compared to the voxelised phantom software,[23] we restricted the analysis to cases with the most commonly used tube voltage cases in each protocol.

### Statistical analysis

We report the estimated median effective dose and organ equivalent doses for the ImPACT and NCICT software and demonstrate the agreement between the software results with Bland-Altman plots. Plots are shown for effective dose (hermaphrodite) by case and organ equivalent dose by median for each protocol for males and females. A typical Bland-Altman plot shows the mean result on the horizontal axis with the difference between the results on the vertical axis with two horizontal lines demonstrating the 95% limits of agreement. These limits of agreement are based on the assumption that the differences are normally distributed, with no relationship between the magnitude of the mean and the difference in results. As this assumption is not met for effective dose, we plot the difference between the methods as a percentage of the NCICT result (i.e. the reference) on the vertical axis.[34, 35] The organ specific median results are also plotted as a proportion. We present the median organ result, as it is the median, rather than individual result that is generally used to estimate cancer incidence using BEIR VII risk coefficients.

The values produced by the ImPACT and NCICT software were also compared using Passing-Bablok regression. Passing-Bablok regression is a robust, non-parametric (i.e. does not rely on assumptions regarding distribution of samples) linear regression test used for method comparison. This regression model reports on the presence of systematic differences (where results vary by a constant amount) and proportional differences (where results vary proportionately) between the method measurements[36]. The model also reports random differences. Random difference between measurement methods is based on the distribution of the remaining variation after correcting for proportional and systematic differences (residuals) Where the Residual Standard Deviation (RSD) interval -1.96 RSD to +1.96 RSD is large, the methods may not be comparable. Only organ categories that contributed at least 10% to the total number of cancers, as averaged across the lifespan (age 18–80) were analysed using Passing-Bablok regression. A linear relationship between the data measurements is required. Where a cumulative sum linearity test (CUSUM) showed deviation from linearity, we have log transformed the data to approximate linearity. For some organs we were unable to approximate linearity and therefore we have not reported the results of the regression.

### Cancer risk modelling

We modelled the estimated lifetime attributable risk (LAR) of cancer inferable from a single exposure at each age separately for males and females for each protocol. To do so, the median specific absorbed organ dose (mGy) (equal to the equivalent dose mSv in the case of CT scanning) of each protocol (male or female) was multiplied with the age-sex specific risk coefficients from Table 12D-1 of the Biologic Effects of Ionizing Radiation (BEIR) VII report [28] to provide the estimated number of cases of cancer per 100,000 individuals exposed at that age. The BEIR VII risk coefficients represent the excess risk that can be directly attributable to a unit (mGy) of ionising radiation. We report LARS for exposure at 18 to 80 years of age, at yearly intervals. This required linear interpolation of the BEIR VII risk coefficients which are reported in 5 yearly intervals to age 20 and then 10 yearly intervals. LAR of cancer incidence was calculated using both the organ dose estimates from the ImPACT software and the NCICT software.

## Results

**Fig 2:**
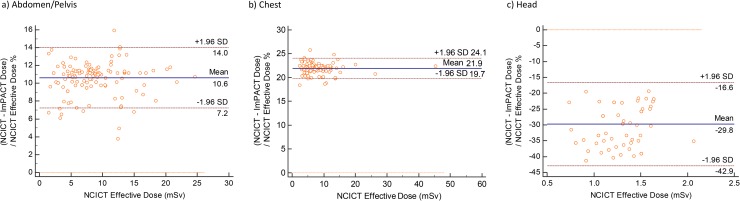
Bland Altman Plots for effective dose for the a) abdomen/pelvis b) chest and c) head protocols.

**Fig 3:**
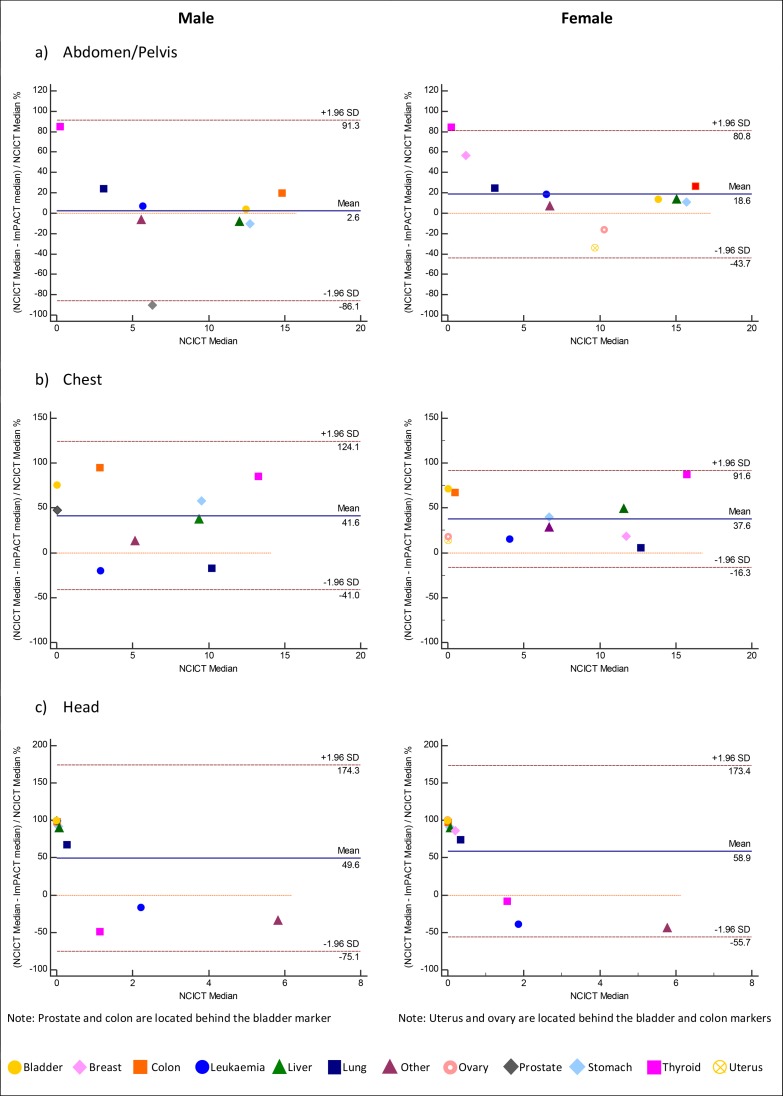
Bland Altman Plots for median organ equivalent dose for the a) abdomen/pelvis b) chest and c) head protocols.

Scanning data were obtained for 160 cases for each of the abdomen/pelvis and head protocols, and 158 cases for the chest protocol. Cases with a tube voltage of 120kV accounted for all of the abdomen/pelvis cases, 155/158 of the chest cases and 124/160 head scan cases. Median effective dose and median organ equivalent dose as calculated by ImPACT and NCICT dosimetry software are shown in Table 1. The relationship between the effective dose as calculated by the ImPACT and NCICT software on a case by case basis are shown in Fig 2, showing consistently larger effective doses calculated by NCICT than ImPACT for the abdomen/pelvis and chest protocols, and lower effective doses for the head protocol. Fig 3 shows the relationship between the ImPACT and NCICT medians for each protocol by male and female. Along the horizontal line is the magnitude of the NCICT median (the reference result), while the vertical line shows the percentage difference between the two estimates relative to the NCICT median. The organs represented below the line of equivalence (i.e. zero difference) are those for which the NCICT median is lower than the ImPACT median. The male ImPACT and NCICT calculations of organ equivalent doses for the abdomen/pelvis protocol show a general decrease in the percentage difference in the estimated organ dose as the magnitude of the dose increases. The greatest percentage difference is shown for the prostate (90%) and the thyroid (84%) dose estimates. However, the thyroid organ receives very little equivalent dose comparatively to the other organs, and in terms of the difference in magnitude, the NCICT dose is only 0.22 mSv higher (Table 1). This is the same for the female thyroid estimates (Table 1). The female results also show large variation for breast (56%) with smallest variation for the stomach (11%) and liver (14%) (Fig 3). The chest protocol (Fig 3B) shows much higher mean variation in results, with the NCICT results that are consistently higher than the ImPACT results, with the exception of leukaemia (i.e. bone marrow) and lung dose estimates in males. The head protocol results (Fig 3C) also show higher NCICT than ImPACT results for most organs.

**Table 1:** Median of the organ equivalent dose for each protocol by BEIR VII category (male and female) and effective dose (gender neutral).

	Abdomen/Pelvis (n = 160)			Chest (n = 155)			Head (n = 124)		
Category (mGy)	ImPACT**	NCICT**	Difference*	ImPACT**	NCICT**	Difference*	ImPACT**	NCICT**	Difference*
**MALE**									
Stomach	14.00	12.70	-1.30	4.00	9.55	5.55	0.00	0.05	0.04
Colon	12.00	14.85	2.85	0.16	2.88	2.72	0.00	0.02	0.02
Liver	13.00	12.04	-0.96	5.80	9.36	3.56	0.01	0.06	0.05
Lung	2.35	3.09	0.74	12.00	10.23	-1.77	0.09	0.27	0.18
Prostate	12.00	6.31	-5.69	0.01	0.02	0.01	0.00	0.00	0.00
Bladder	12.00	12.48	0.48	0.01	0.04	0.03	0.00	0.00	0.00
Other	5.91	5.57	-0.34	4.45	5.15	0.70	7.80	5.83	-1.98
Thyroid	0.04	0.26	0.22	2.00	13.29	11.29	1.70	1.14	-0.56
Leukaemia	5.30	5.69	0.39	3.50	2.91	-0.59	2.60	2.22	-0.38
**FEMALE**									
Stomach	14.00	15.70	1.70	4.00	6.68	2.68	0.00	0.04	0.04
Colon	12.00	16.30	4.30	0.16	0.48	0.32	0.00	0.01	0.01
Liver	13.00	15.07	2.07	5.80	11.57	5.77	0.01	0.06	0.06
Lung	2.35	3.10	0.75	12.00	12.70	0.70	0.09	0.34	0.25
Breast	0.51	1.17	0.66	9.60	11.73	2.13	0.03	0.19	0.17
Uterus	13.00	9.68	-3.32	0.04	0.05	0.01	0.00	0.01	0.01
Ovary	12.00	10.29	-1.71	0.05	0.06	0.01	0.00	0.01	0.01
Bladder	12.00	13.88	1.88	0.01	0.04	0.03	0.00	0.01	0.00
Other	6.21	6.70	0.48	4.72	6.65	1.93	8.26	5.77	-2.49
Thyroid	0.04	0.26	0.22	2.00	15.74	13.74	1.70	1.57	-0.13
Leukaemia	5.30	6.50	1.20	3.50	4.11	0.61	2.60	1.86	-0.74
**GENDER NEUTRAL**									
Effective dose (mSv)	6.70	7.51	0.81	5.10	6.49	1.39	1.80	1.32	-0.48

* Difference equals ImPACT median subtract NCICT median

** Shaded cells are those organs that contribute on average at least 10% of the total number of cancers across the lifespan as calculated using the BEIR VII Lifetime attributable risk coefficients for ages 18 to 80.

The organs that contribute at least 10% to average cancer incidence over the lifespan are shown in the shaded cells in Table 1. There were significant, although often-times small, proportional differences between the ImPACT and NCICT equivalent dose estimates for both males and females in all protocols for all these organs (excluding those for which linearity could not be approximated) (Table 2). Many organs also showed small significant systematic differences. A systematic difference is one where the results differed by a constant amount. In evaluating Passing Bablok regression, random differences should be reported as this demonstrates if the methods are comparable. In our study no significant random differences were identified (Table 2 confidence intervals cross zero), suggesting the methods are comparable.

**Table 2:** Passing Bablok regression–Comparison of median organ equivalent doses as estimated by ImPACT and NCICT for each protocol (abdomen pelvis, chest and head) for those organs that contribute >10% to total cancer incidence (averaged over ages 18–80) for a) males and b) females.

	% contribution^a^		Regression equation^b^	Differences (95% CI)		
Organ	ImPACT	NCICT	ImPACT = x, NCICT = y	Systematic^c^	Proportional^d^	Random^e^
Male						
**Abdomen Pelvis**						
Bladder	19	19	y = -0.11 + 1.06x	-0.1133 (-0.19, -0.01)	1.0589 (1.04, 1.07)	0.30 (-0.59, 0.59)
Colon	28	33	log(y) = 0.09 + 1.01 log(x)	0.0934 (0.09, 0.10)	1.0063 (1.00, 1.01)	0.01 (-0.01, 0.01)
Other	19	17	y = -0.02 + 0.95 x	-0.020 (-0.04, 0.003)	0.95 (0.95, 0.96)	0.08 (-0.15, 0.15)
**Chest**						
Colon	<1	11	y = 0.18 + 16.17 x	0.18 (0.11, 0.21)	16.17 (15.81, 16.74)	0.02 (-0.03, 0.03)
Lung	50	36	y = -0.14 + 0.84 x	-0.14 (-0.21, -0.03)	0.84 (0.83, 0.84)	0.23 (-0.45, 0.45)
Other	28	28	Cannot approx. linearity^f^			
**Head**						
Leukaemia	83	80	y = -0.13 + 0.95 x	-0.13 (-0.22, -0.03)	0.95 (0.90, 0.99)	0.10 (-0.19, 0.19)
Other	15	17	Cannot approx. linearity^f^			
Female						
**Abdomen Pelvis**						
Bladder	18	18	y = -0.13 + 1.18 x	-0.13 (-0.21, -0.02)	1.18 (1.16, 1.19)	0.32 (-0.62, 0.62)
Colon	18	21	Log(y) = 0.13 + 1.01 log(x)	0.13 (0.13, 0.14)	1.01 (1.00, 1.01)	0.01 (-0.01, 0.01)
Lung	11	13	y = -0.01 + 1.32 x	-0.012 (-0.03, -0.00)	1.32 (1.31, 1.33)	0.05 (-0.09, 0.09)
Other	21	19	y = -0.018 + 1.09 x	-0.02 (-0.05, 0.01)	1.09 (1.08, 1.10)	0.09 (-0.17, 0.17)
**Chest**						
Breast	25	25	y = 0.16 + 1.17 x	0.16 (0.08, 0.31)	1.17 (1.16, 1.18)	0.28 (-0.55, 0.55)
Lung	52	44	y = -0.17 + 1.04 x	-0.17 (-0.26, -0.03)	1.04 (1.03, 1.04)	0.25 (-0.50, 0.50)
Other	14	16	y = -0.06 + 1.43 x	-0.06 (-0.10, -0.01)	1.43 (1.42, 1.44)	0.09 (-0.17, 0.17)
**Head**						
Leukaemia	86	78	y = -0.12 + 0.80 x	-0.12 (-0.19, 0.01)	0.80 (0.74, 0.83)	0.09 (-0.18, 0.18)
Other	10	10	Cannot approx. linearity^f^			

^a^ Proportion of the total number of cancers across the lifespan as calculated using the BEIR VII Lifetime attributable risk coefficients for ages 18 through 80.

^b^ Regression equation: the regression equation with the calculated values for intercept A and slope B according to Passing & Bablok (1983). The equation converts the dose calculated by ImPACT (x) to a new dose calculated by NCICT (y)

^c^ Systematic differences (intercept A): a measure of the systematic differences between the two methods. The 95% confidence interval for the intercept A tests the hypothesis that A = 0. This hypothesis is accepted if the confidence interval for A contains the value 0. If the hypothesis is rejected, then it is concluded that A is significantly different from 0 and the methods differ by a constant amount. Significant differences are shown in shaded cells.

^d^ Proportional differences (slope B): a measure of the proportional differences between the two methods. The 95% confidence interval for the slope B tests the hypothesis that B = 1. This hypothesis is accepted if the confidence interval for B contains the value 1. If the hypothesis is rejected, then it is concluded that B is significantly different from 1 and there is a proportional difference between the two methods. Significant differences are shown in shaded cells.

^e^ Random differences (residual standard deviation RSD): a measure of the random differences between the two methods. 95% of random differences are expected to lie in the interval -1.96 RSD to +1.96 RSD. If this interval is large, the two methods may not be comparable. Significant differences are shown in shaded cells.

^f^ Linear model validity: the CUSUM test for linearity is used to evaluate how well a linear model fits the data. Where p<0.05 there is significant deviation from linearity. Passing Bablok regression assumes linearity, therefore results are not shown.

**Fig 4:**
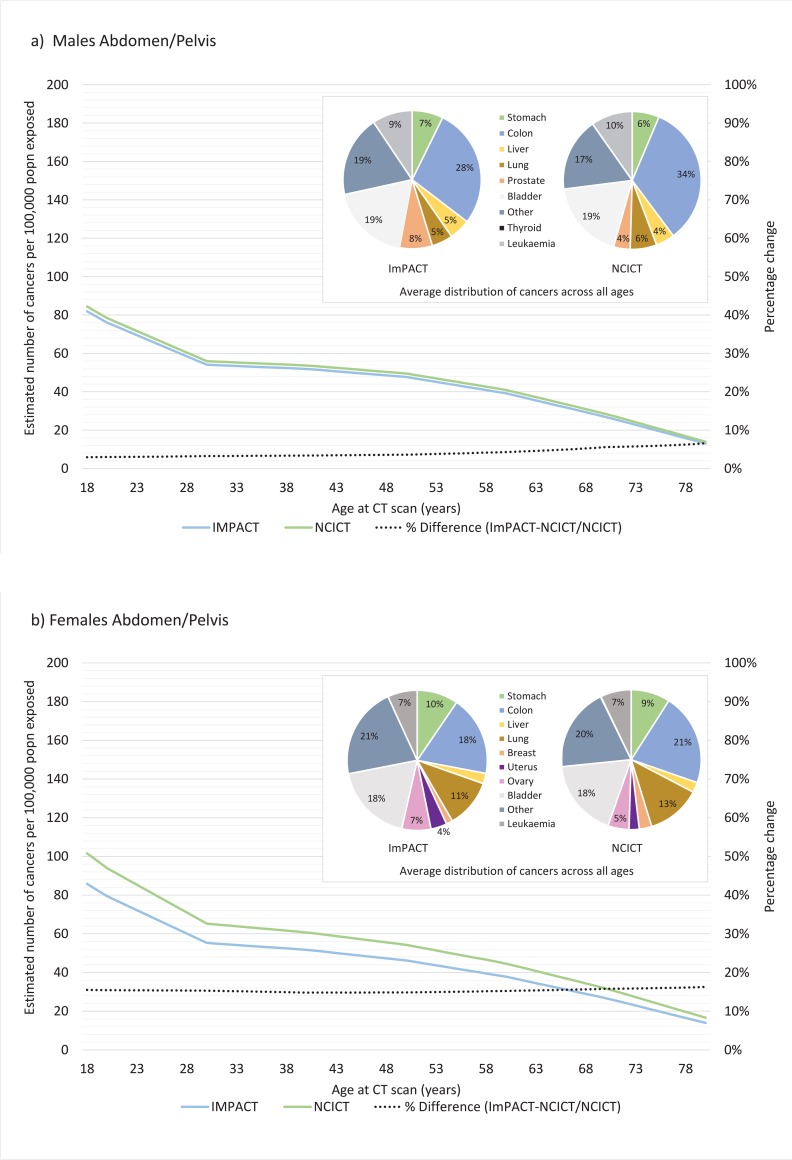
Lifetime attributable risk of cancer incidence for a) males and b) females exposed to radiation associated with an abdomen pelvis CT scanning protocol as calculated using ImPACT or NCICT software. Figure inset shows the average distribution of cancers across the lifespan (18–80 years) for each type of software (percentage contribution <4% are not annotated).

**Fig 5:**
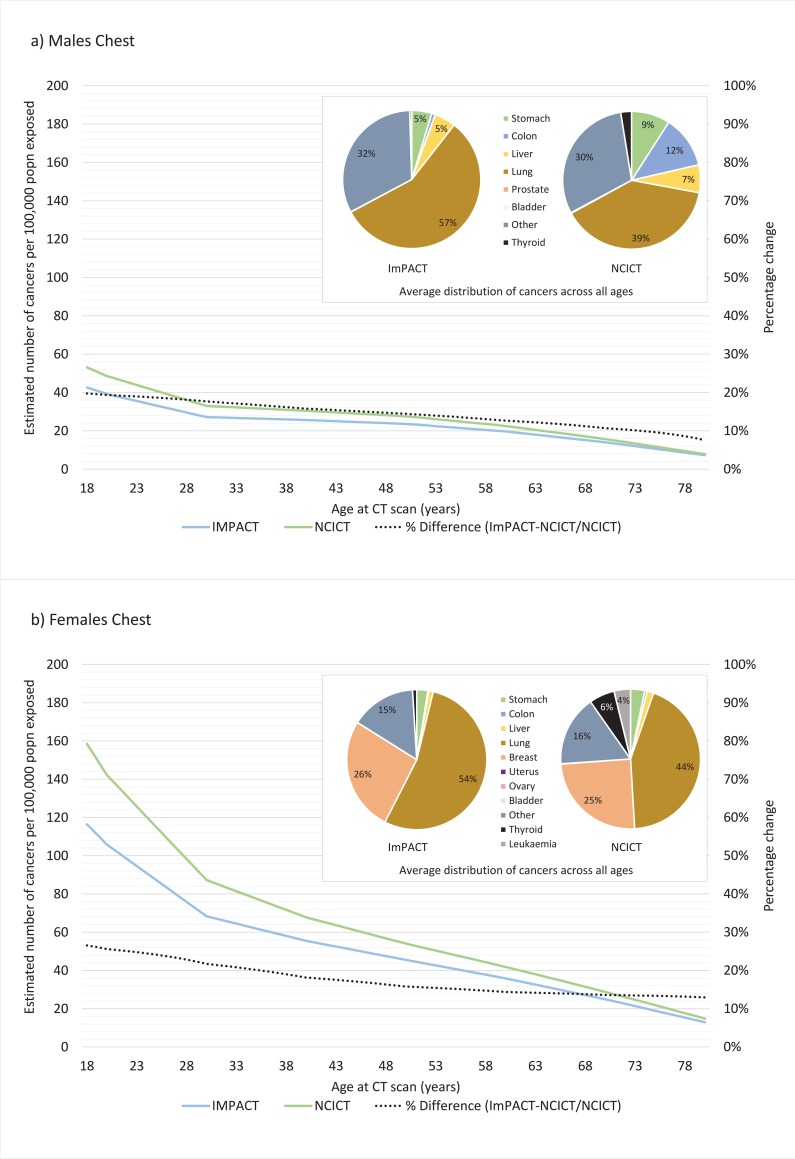
Lifetime attributable risk of cancer incidence for a) males and b) females exposed to radiation associated with a chest CT scanning protocol as calculated using ImPACT or NCICT software. Figure inset shows the average distribution of cancers across the lifespan (18–80 years) for each type of software (percentage contribution <4% are not annotated).

**Fig 6:**
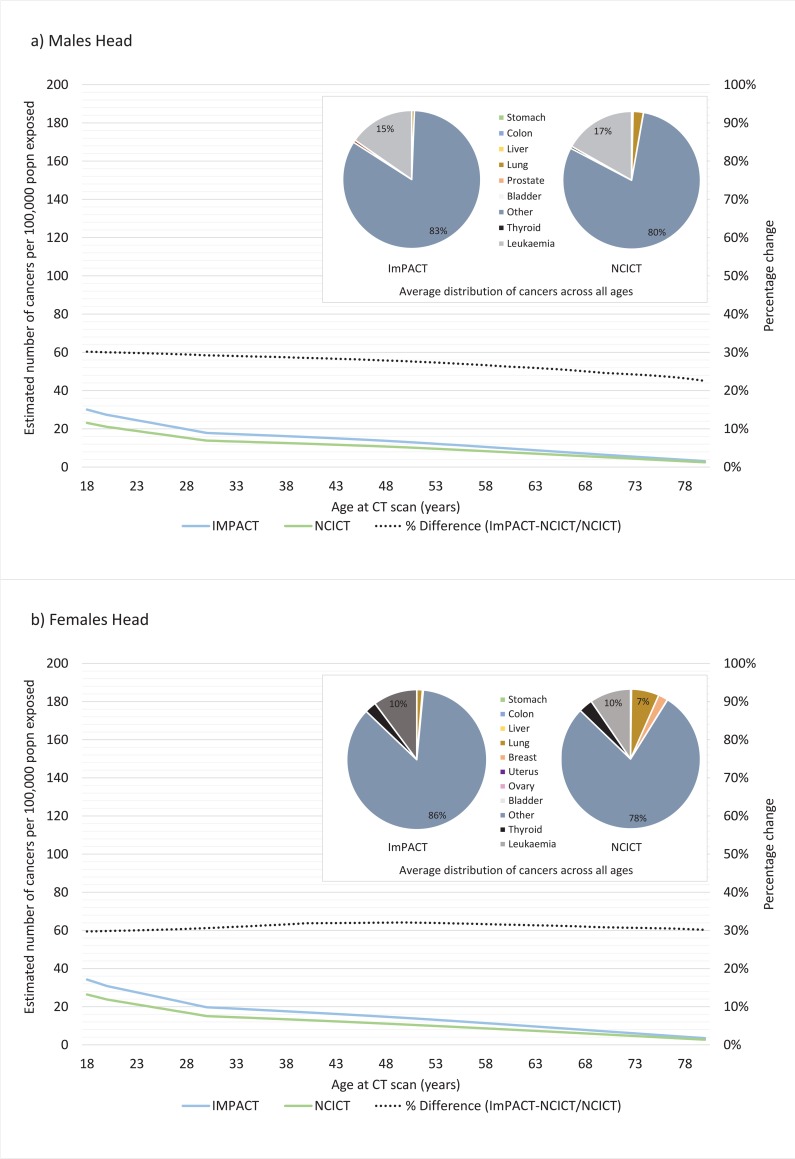
Lifetime attributable risk of cancer incidence for a) males and b) females exposed to radiation associated with a head CT scanning protocol as calculated using ImPACT or NCICT software. Figure inset shows the average distribution of cancers across the lifespan (18–80 years) for each type of software (percentage contribution <4% are not annotated).

The contribution of exposure to the CT scanning protocol on cancer incidence across the lifespan is shown in Figs 4–6 using both the ImPACT and NCICT organ equivalent medians for males and females. With the exception of the head protocol (Fig 6), in all cases, the NCICT dosimetry estimates result in larger estimates of cancer incidence. This difference is more pronounced for females than males. The inset pie charts generally show a similar distribution of cancers over the lifespan as a result of CT scanning for results generated from ImPACT compared to NCICT dosimetry estimates for all protocols. The chest protocol shows the most variation, with some difference in the proportion of cancer attributed to lung, colon and thyroid cancer.

## Discussion

For the majority, but not all, of the organs used in epidemiological studies of cancer incidence, the NCICT software (voxelised phantom) provides higher estimates of organ dose among three different scanning protocols compared to the ImPACT software (mathematical phantom). The smallest percentage differences were generally seen among those organs that are included in their entirety in the scan region. These are stomach, liver, bladder, colon, uterus and ovaries for the abdomen/pelvis protocol and lung and breast for the chest protocol, with larger percentage differences seen for organs further away, or on the boundary of the scan region. The head protocol, for which most of the organs considered were outside the scan region, the percentage difference between the software results are highest. This is a general observation, and some deviation does occur. Within the chest protocol among females, the uterus and ovaries (outside of the scan region) had percentage differences equal to or smaller than the within-scan organs, breast and lung. These results showing greater percentage differences depending on the proximity of the organ to the scan region are consistent with previous studies comparing software using mathematical and voxelised phantoms.[22, 27]

The differences in the results may be largely explained by the anatomical variation between the phantoms with estimated radiation dose sensitive to the shape, size and position of the organ.[22] As stated previously, consistency of the scan positions between the phantoms was challenging due to the anatomical, size and/or postural variation. This is likely to have had greatest impact on the boundary organs. Direct comparison with the values of the results from other studies comparing software using voxelised and mathematical phantoms are likely to be meaningless due to these factors as well as differences in scanning parameters, and other characteristics of the software. Through linear regression modelling we present formulae to convert the results of the two software packages, however it is evident that this is an impractical application for the reasons provided above. While the results of the two different software programs are comparable (i.e. the residual standard deviation was low), the models differ between organs and between protocols. These conversion formulae are likely to apply only to these very specific scan regions, parameters and specific software used in our study.

Software programs based on mathematical phantoms are still available and estimates of the lifetime attributable risk of cancer following CT radiation have relied on organ dose estimates calculated from dosimetry software based on these mathematical phantoms as recently as 2015. [2, 7, 8, 11, 12, 37] This is despite the 2009 ICRP recommendation to use voxelised phantoms.[20] Our data demonstrate disparity in estimated cancer incidence with NCICT (voxelised phantom) calculated doses resulting in cancer estimates 2.9% to 6.6% higher in males and 14.8% to 16.3% higher in females for the abdomen/pelvis protocol across the lifespan. This disparity was greater for the chest protocol, with NCICT (voxelised) cancer incidence estimates 7.6% to 19.7% higher in males and 12.9% to 26.5% higher in females compared to ImPACT. The head protocol was the only protocol for which the overall cancer incidence estimates were lower for the NCICT (voxelised) software, but had the greatest disparity in results—in excess of 30% at times. This can be attributed to the much higher dose estimate for bone marrow in the ImPACT software, with leukaemia accounting for approximately 80% of all cancers resulting from the CT scan.

The extent to which these radiation dose estimates impact the results must be considered within context. Uncertainty exists not only in dose assessment, but, perhaps more so, within the data on the health effects of radiation exposure. Coefficients used to quantify the risk of cancer as a result of CT radiation exposure are subject to revision and based on epidemiological studies that have inherent limitations. The BEIR VII risk coefficients used in the current study, as with other sources,[21] have largely been derived from Japanese atomic bomb survivors, supported by smaller studies on health effects among those exposed to radiation medically and occupationally.[21, 28] The characteristics of these studied populations and their radiation exposure are unlikely to be directly transferable to the population of interest. As an example, baseline risks for many cancer sites in the United States, for which the BEIR VII coefficients have been adapted, are substantially different to those in Japan, impacting the attribution of risk. Furthermore, exposure among atomic bomb survivors, as well as in other studies, has often been at high doses, unlike the low dose of CT.[28] As a result assumptions have to be made about “exactly how radiation exposure increases the risk of cancer” and how to transfer risk between populations.[28] The reliance on assumptions and subjective opinion has led to healthy debate about these data. [38, 39] Many more sources of uncertainty, and examples, in the risk estimates for radiation induced cancer have been described.[40]

Table 12–13 on page 291 of the BEIR VII report provides subjective confidence intervals around the whole body cancer risk from radiation exposure. In males, it is estimated that there are 800 excess cases of cancer (all solid cancer) from exposure to 0.1 Gy, with subjective confidence intervals of 400 and 1600. In females for the same exposure, excess cases are 1300 (CI, 690, 2500). As a ratio, the subjective confidence intervals for leukaemia are broader, with a point estimate of 100 (CI 30, 300) for males and 70 (20, 250) for females.[28] If we consider these ratios to apply to our results, the real cancer incidence resulting from our estimated radiation doses may be between a third and threefold of those estimated for leukaemia, or between half and twofold of those estimated for all solid cancers. However, the percentage difference between the estimates of cancer incidence resulting from each software program remains the same regardless of the cancer risk coefficients. It is arguable that the uncertainty in health effects of radiation exposure surpasses any concerns regarding the disparity between the software dose estimates.

In addition to the uncertainty of these risk estimates, this study has a number of limitations related to assumptions made about anatomical position, the required manipulation of data to normalise CTDIvol, scan length variation and the assumptions made to categorise organs into “other” in ImPACT compared to NCICT. It is evident from the literature, and these limitations, that any attempt to quantify population cancer incidence attributable to radiation exposure is subject to great uncertainty. For this reason, cancer incidence estimates may be more meaningful for relative comparison within studies in which assumptions and dosimetry software are consistent. What may be most important in the literature, other than how dose is estimated, is that methodology is described comprehensively and consistently to improve transparency, reproducibility and scientific value, with clear indication of the type of software and phantom used for dose estimates. Reporting guidelines have been developed for these reasons in other areas, such as CONSORT for randomised trials or RECORDS for Monte Carlo radiation transport studies.[41] Comparison with other studies should also be undertaken with care and overall uncertainty must be emphasised. In terms of policy implications, our results suggest lower cancer incidence than previously thought as a result of head scans, but higher incidence—more so for females—for both abdomen/pelvis and chest scans.

## Conclusion

Previous studies estimating cancer incidence using mathematical phantoms remain valid. However when the results of these studies are compared to more recent, or future, studies using voxelised and/or hybrid phantoms, the role of different software in the calculation of results must be considered. Definite comparisons should only be made where dosimetry and cancer estimates can be recalculated using the same software. Where this is not feasible, care must be taken not to overlook the potential role of the change in software on outcomes. It is clear that any change, or lack of change, in radiation dose or cancer risk may be attributable to the type of software and phantom used, rather than—or in addition to—changes in scanning practice. In line with the recommendations of the ICRP, new studies should aim to use software based on the more anatomically realistic voxelised phantoms. Studies using dosimetry software to estimate radiation dose should describe the software comprehensively to facilitate comparison with both past and future research.

## Supporting information

S1 Table ImPACT and NCICT organ categories matched with BEIR VII category. (DOCX)

S1 Fig Male dosimetry estimates. (DOCX)

S2 Fig Female dosimetry estimates. (DOCX)
