# Supplementary material for: How have advances in CT dosimetry software impacted estimates of CT radiation dose and cancer incidence? A comparison of CT dosimetry software: Implications for past and future research
Source: PLoS One. 2019 Aug 14;14(8):e0217816. doi: 10.1371/journal.pone.0217816 (PMC6693687; doi:10.1371/journal.pone.0217816)
Supplement: S1 Table — (DOCX) [file pone.0217816.s001.docx]

**S1 Table. ImPACT and NCICT organ categories matched with BEIR VII category.**

| **MALE** | | | **FEMALE** | | |
| --- | --- | --- | --- | --- | --- |
| **BEIR Categories** | **NCICT** | **IMPACT** | **BEIR Categories** | **NCICT** | **IMPACT** |
| Stomach | Stomach wall | Stomach | Stomach | Stomach wall | Stomach |
| Colon | Colon | Colon | Colon | Colon | Colon |
| Liver | Liver | Liver | Liver | Liver | Liver |
| Lung | Lungs | Lung | Lung | Lungs | Lung |
| Bladder | Urinary bladder | Bladder | Bladder | Urinary bladder | Bladder |
| Thyroid | Thyroid | Thyroid | Thyroid | Thyroid | Thyroid |
| Leukaemia | Active marrow | Bone marrow | Leukaemia | Active marrow | Bone marrow |
| Prostate | Prostate | Prostate | Breast | Breast | Breast |
|  |  |  | Uterus | Uterus | Uterus |
|  |  |  | Ovary | Ovaries | Ovaries |
| Other* | Brain | Brain | Other* | Brain | Brain |
|  | Salivary glands | Salivary glands |  | Salivary glands | Salivary glands |
|  | Adrenals | Adrenals |  | Adrenals | Adrenals |
|  | Small intestine | Small intestine |  | Small intestine | Small intestine |
|  | Kidney | Kidney |  | Kidney | Kidney |
|  | Spleen | Spleen |  | Spleen | Spleen |
|  | Skin | skin |  | Skin | skin |
|  | Muscle | Muscle |  | Muscle | Muscle |
|  | Gall bladder | Gall bladder |  | Gall bladder | Gall bladder |
|  | Pancreas | Pancreas |  | Pancreas | Pancreas |
|  | Thymus | Thymus |  | Thymus | Thymus |
|  | Esophagus | Oesophagus |  | Esophagus | Oesophagus |
|  | Oral cavity | Oral mucosa |  | Oral cavity | Oral mucosa |
|  | Heartwall | heart |  | Heartwall | heart |
|  | Shallow marrow | Bone surface |  | Shallow marrow | Bone surface |
|  | Trachea | ET region |  | Trachea | ET region |
|  | Spinal cord | Lymph nodes |  | Spinal cord | Lymph nodes |
| * The absorbed dose value for 'Other' is the mean of the median doses for the listed organs. | | | | | |
