# Supplementary material for: How have advances in CT dosimetry software impacted estimates of CT radiation dose and cancer incidence? A comparison of CT dosimetry software: Implications for past and future research
Source: PLoS One. 2019 Aug 14;14(8):e0217816. doi: 10.1371/journal.pone.0217816 (PMC6693687; doi:10.1371/journal.pone.0217816)
Supplement: S2 Fig — (DOCX) [file pone.0217816.s003.docx]

**S2 Fig Female Dosimetry Estimates** – **Bland Altman Plots of organ dose by protocol**

| **Female Dosimetry Estimates – Organ Doses NCICT and ImPACT estimates by Protocol***  *Organ doses presented are those organs that contribute >10% of estimated cancers over a lifetime as per BEIR VII calculations | | |
| --- | --- | --- |
| **Abdomen/Pelvis Protocol – Colon** | **Abdomen/Pelvis Protocol – Bladder** | **Abdomen/Pelvis Protocol – Lung** |
| 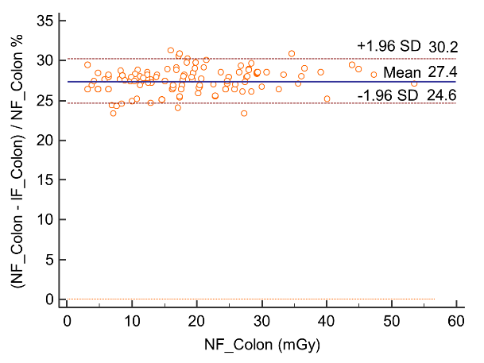 |  | 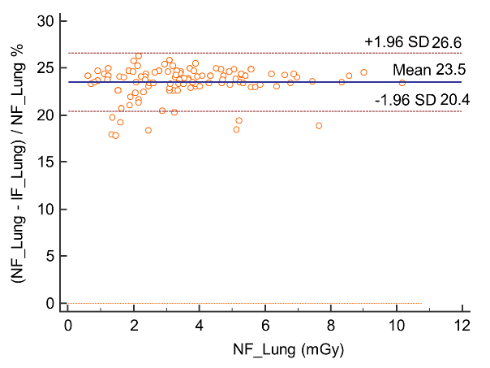 |
| **Abdomen/Pelvis Protocol – Other** | **Chest Protocol – Lung** | **Chest Protocol – Breast** |
|  |  |  |
| **Chest Protocol – Other** | **Head Protocol – Other** | **Head Protocol – Leukaemia** |
| 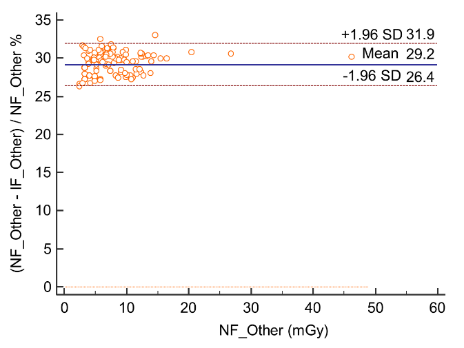 |  |  |
| NF: NCICT Female estimate, IF: ImPACT Female estimate | | |
